# 

**Published:** 1911-04-15

**Authors:** 


					﻿Dental
Register
NELVI.LLE S. HOFF, Editor,
Ann Arbor, iVlich.
Vol. LXV. —APRIL, 1911— No. 4
SAMUEL A. CROCKER & CO., Publishers
OHIO DENTAL AND SURGICAL DEPOT
35 W. FIFTH STREET, CINCINNATI, 0.
PRICE, ONE DOLLAR A YEAR
Entered at the Postoffice at Cincinnati, 0., as second-class mail matter.
				

